# *Pyricularia oryzae* enhances *Streptomyces griseus* growth via non‐volatile alkaline metabolites

**DOI:** 10.1111/1758-2229.70012

**Published:** 2024-09-23

**Authors:** Risa Sugiura, Takayuki Arazoe, Takayuki Motoyama, Hiroyuki Osada, Takashi Kamakura, Kouji Kuramochi, Yuuki Furuyama

**Affiliations:** ^1^ Department of Applied Biological Science, Faculty of Science and Technology Tokyo University of Science Noda‐shi Japan; ^2^ Plant Immunity Research Group RIKEN Center for Sustainable Resource Science (CSRS) Wako‐shi Japan; ^3^ Institute of Microbial Chemistry Shinagawa‐ku Japan

## Abstract

Chemical compounds that affect microbial interactions have attracted wide interest. In this study, *Streptomyces griseus* showed enhanced growth when cocultured with the rice blast fungus *Pyricularia oryzae* on potato dextrose agar (PDA) medium. An improvement in *S. griseus* growth was observed before contact with *P. oryzae* , and no growth‐promoting effect was observed when the growth medium between the two microorganisms was separated. These results suggested that the chemicals produced by *P. oryzae* diffused through the medium and were not volatile. A PDA plate supplemented with phenol red showed that the pH of the area surrounding *P. oryzae* increased. The area with increased pH promoted *S. griseus* growth, suggesting that the alkaline compounds produced by *P. oryzae* were involved in this growth stimulation. In contrast, coculture with the soilborne plant pathogen *Fusarium oxysporum* and entomopathogenic fungus *Cordyceps tenuipes* did not promote *S. griseus* growth. Furthermore, DL‐α‐Difluoromethylornithine, a polyamine biosynthesis inhibitor, prevented the increase in pH and growth promotion of *S. griseus* by *P. oryzae* . These results indicated that *P. oryzae* increased pH by producing a polyamine.

## Graphical abstract

The growth of *Streptomyces griseus* was promoted on potato dextrose agar (PDA) medium when cocultured with *Pyricularia oryzae*, but not when cultured alone. A PDA plate supplemented with phenol red indicated that *P. oryzae* increased the pH of the surrounding area. The area with increased pH promoted *S. griseus* growth, suggesting that the alkaline compounds produced by *P. oryzae* stimulated this growth.

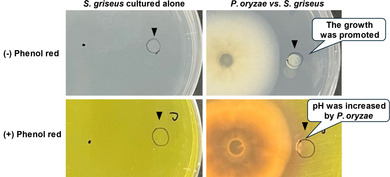

## INTRODUCTION

Fungi and bacteria interact with each other and build a complex community in natural environments. However, conventional microbiology relies on studies with pure culture, which leads to behavioural observations that differ from the natural state (Pierce et al., 2021). Microbial behaviour can be influenced by neighbouring organisms, leading to various consequences, such as modification of virulence of human pathogens (Santus et al., 2021), biofilm formation (Rodrigues et al., 2020), growth improvement (Jones et al., 2017), and increases in the production of specialized metabolites (Fischer et al., 2018.; Netzker et al., 2015; Stubbendieck & Straight, 2016). Therefore, a thorough understanding of microbial ecology in the natural environment is necessary to fully exploit the potential of useful microorganisms and develop efficient methods for controlling pathogens. This requires the elucidation of molecular‐level interactions among various microorganisms. However, identifying specific species of microorganisms involved in such interactions and the signalling molecules exchanged among them remains a great challenge.

The rice blast fungus, *Pyricularia oryzae* (syn. *Magnaporthe oryzae*), is a model plant pathogenic fungus that causes the most devastating disease of rice worldwide (Dean et al., 2005). The blast fungi spread through airborne conidia and colonize the leaves and panicles of host plants (Fernandes et al., 2017). To control blast disease, a detailed understanding of the ecology of this fungus in natural environments is necessary; however, most studies regarding this fungus have focused on its infection behaviour, and the ecology of this fungus outside plants, such as in soil, remains unknown. Some studies suggest that blast fungi are distributed in soil (Marcel et al., 2010; Sesma & Osbourn, 2004). Multitudes of microorganisms interact in soil. Therefore, soil‐residing *P. oryzae* may also interact with other microorganisms. However, reports analysing the interactions between this fungus and other microorganisms are limited.

*S. griseus* is a soil‐dwelling bacterium and primarily produces secondary metabolites, including antibiotics, such as cycloheximide (Fischer et al., 2018.; Netzker et al., 2015; Pittenger & McCoy, 1953). Secondary metabolites produced by actinomycetes are used in medicine and agriculture. Antibiotics and volatile organic compounds play an important role in biologically controlling phytopathogenic microorganisms and inhibiting bacterial quorum sensing (Wang et al., 2024). Furthermore, actinomycetes live in plants and induce disease resistance by expressing resistance‐associated genes in the host and accumulating phytoalexins (Ansari et al., 2020). *Streptomyces* spp. effectively suppress the onset of rice blast disease when simultaneously inoculated with *P. oryzae* (Law et al., 2017); this report indicates that *Streptomyces* spp. interact with *P. oryzae* in rice plants; however, the mechanisms underlying this interaction have not been fully elucidated.

*P. oryzae* and *S. griseus* compete with each other by producing secondary metabolites that exhibit antimicrobial activity (Furuyama et al., 2021). Therefore, the present study aimed to identify the mechanism of interaction between these two species in a laboratory setting.

## EXPERIMENTAL PROCEDURES

### Strains, medium, and culture conditions

*P. oryzae* Hoku1, Kyu89‐246, and P2 isolates, *Fusarium oxysporum* f. sp. *lycopersici* MAFF103036 isolate, and *Cordyceps tenuipes* TUS‐1 isolate were used in this study. Fungi were grown on Potato Dextrose Agar (PDA; Difco Co., Franklin Lakes, NJ, USA) plates. The confrontation culture was grown on PDA plates at 28°C. In some studies, phenol red was added to PDA (final concentration: 0.0025%); and DL‐α‐Difluoromethylornithine (DMFO) (Nacalai Tesque Inc., Kyoto, Japan) was added (final concentration: 0.1, 0.5, and 1.0 mM). *S. griseus* (NBRC13350) was used as a competitor. This bacterium was grown on a yeast extract agar (YSA; 0.2% yeast extract, 1% soluble starch, 1.5% agar, pH 7.3) plates at 28°C. Spores scraped from the plates were suspended in sterile distilled water and collected by centrifugation at 4000 × *g* for 5 min at 25°C. The pH of the media was measured using a LAQUAact pH meter (HORIBA, Ltd., Kyoto, Japan).

### Confrontation culture

The confrontation culture was grown on 90‐mm PDA plates as previously described (Furuyama et al., 2021). In brief, spore solutions (10 μL, 1.0 × 10^8^ cells/mL) of *S. griseus* and agar plugs of precultured *P. oryzae*, *F. oxysporum*, or *C. tenuipes* were inoculated to PDA plates and incubated at 28°C for 7–29 days; the initial distance of bacteria and fungi were 2, 2, and 1 cm, respectively. The medium between *P. oryzae* and *S. grisea* (2 mm) was removed using a spatula to separate their growth zones.

### Extraction and detection of ammonia

To extract ammonia, *P. oryzae* was cultured on filter paper and placed on phenol red‐supplemented PDA. After the media turned red (approximately 15 dpi), *P. oryzae* mycelia were removed using filter paper. The PDA media was then hollowed out in six places using sterile straws. Each agar fragment was placed in a 15 mL tube and extracted overnight at 4°C in 1.5 mL Milli‐Q water. The supernatant was used as ammonia extract and ammonia was detected using a modified indophenol method (Nishikawa, 2023). An ammonia solution (28%) (Nacalai Tesque Inc., Kyoto, Japan) was serially diluted in a half‐log dilution series covering a range of 10^−3^ to 1/8 × 10^−3^ and used as the standard solution. An aqueous solution of sodium hypochlorite was diluted 15 times and used as solution 1. A 1.0 M aqueous solution of sodium hydroxide was used as solution 2. Next, 1.2 M sodium salicylate (FUJIFILM Wako Pure Chemicals, Tokyo, Japan), 26 mM potassium hexacyanoferrate(II) trihydrate (FUJIFILM Wako Pure Chemicals), and 20 mM ethylenediaminetetraacetic acid disodium salt dihydrate (Nacalai Tesque Inc.) were mixed to prepare solution 3. Solutions 1–3 (500 μL each) were mixed in a sample tube; 500 μL standard ammonia solution or water extract was added to the mixture, and the reaction was performed at 25°C. After a light blue colour appeared in the sample tube containing standard ammonia solution, 200 μL reaction mixture from each sample tube was transferred to a 96‐well plate, and the optical density was measured at 635 nm.

### Statistical analysis

All experimental results are expressed as the mean ± standard deviation of three replicates. Differences were analysed using Dunnett's test to determine differences between experimental samples (triplicate) using Microsoft Office Excel and R software (version 4.0.2, R Foundation for Statistical Computing, Austria).

## RESULTS

### P. oryzae promotes the growth of S. griseus

**FIGURE 1:**
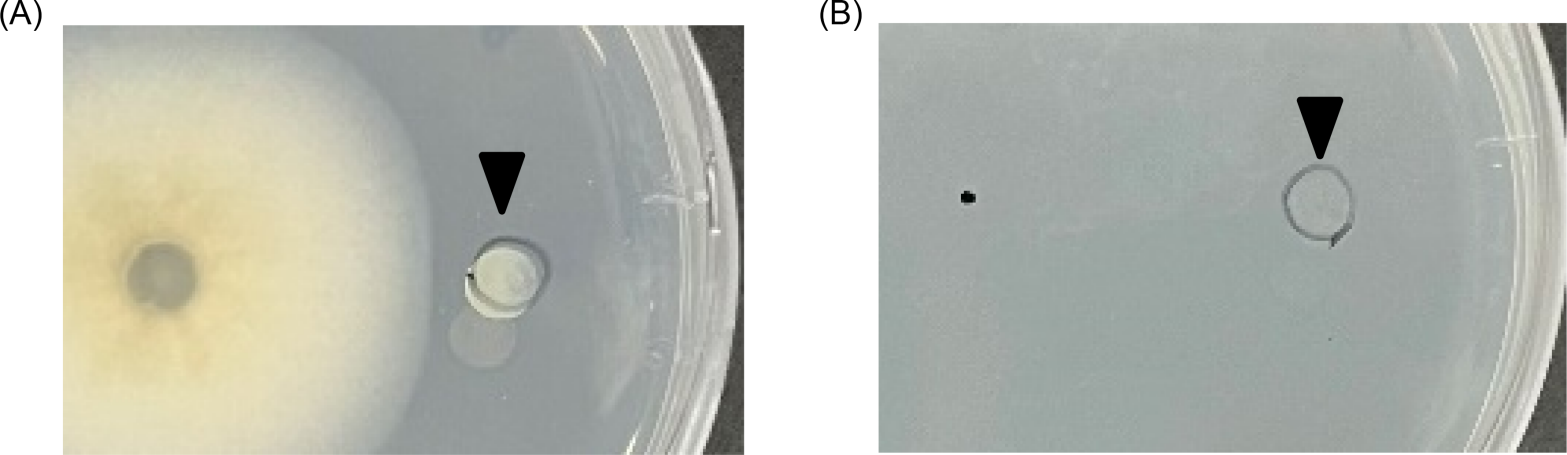
Photographs showing the growth promotion of *Streptomyces griseus* by *Pyricularia oryzae*. (A) Confrontation culture of *P. oryzae* (left colony) and *S. griseus* (right colony). (B) *S. griseus* cultured alone at pH 4.8 showing no growth. Images were captured at 6 dpi. The black triangle indicates the point of inoculation of *S. griseus* .

The growth of *S. griseus* was promoted by confrontation with *P. oryzae* on PDA medium (Figure 1A); however, when cultured alone, *S. griseus* did not grow under the same conditions (Figure 1B). Growth induction was observed at 6 days post‐inoculation (dpi) when the distance between the two microorganisms on the plate was approximately 5 mm (Figure 1A). Because the growth of *S. griseus* was affected without physical contact, metabolites produced by *P. oryzae* might be involved in this phenomenon.

### pH of the medium is important for the growth of S. griseus

**FIGURE 2:**
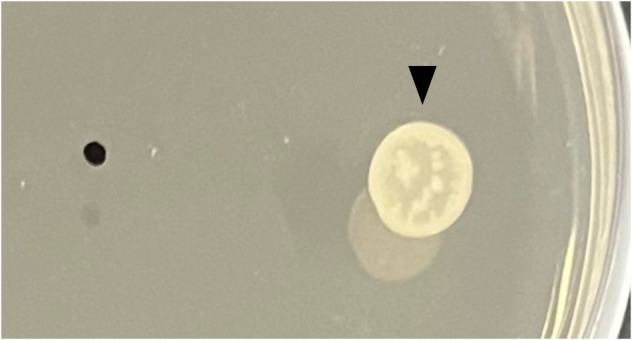
Photograph showing the growth of *Streptomyces griseus* when the pH of the PDA medium was adjusted to 7.5. On the neutralized PDA, *S. griseus* could grow without *Pyricularia oryzae*. The image was captured at 1 dpi. The black triangle indicates the point of inoculation of *S. griseus* .

In the present study, the pH of the PDA medium was 4.8, although the optimal pH for the growth of *S. griseus* is 7.5. Therefore, we speculate that the low pH condition inhibited the growth of *S. griseus*. The pH of the PDA medium adjusted to 7.5 resulted in the growth of *S. griseus* even as a single culture (Figure 2). This result led us to consider that the pH around the *S. griseus* colony was affected by compounds produced by *P. oryzae*.

### Changes in the pH of the medium by P. oryzae induces the growth of S. griseus

**FIGURE 3:**
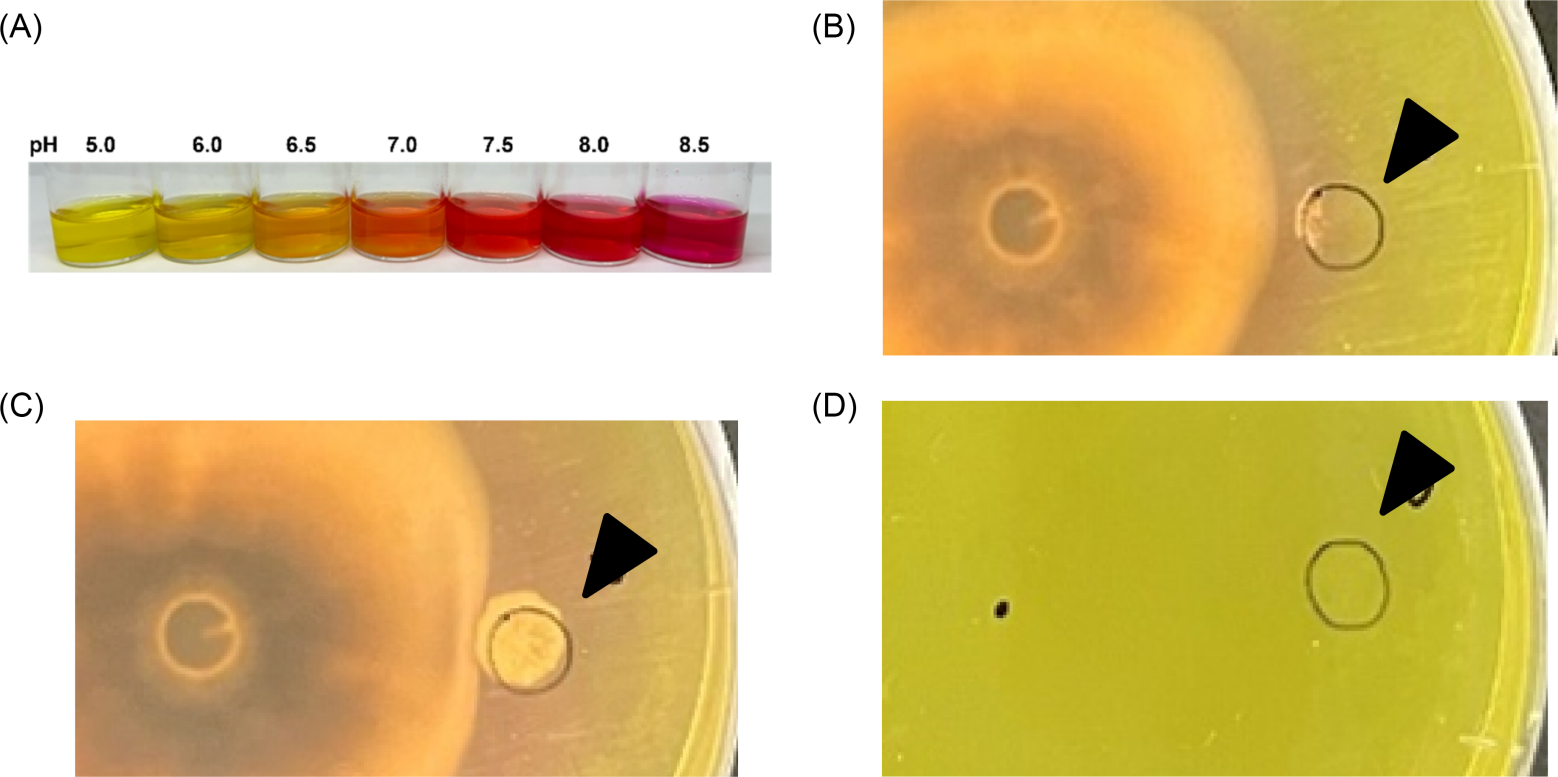
Photographs showing the growth of *Pyricularia oryzae* with increasing medium pH. (A) Relationship between pH of media and colour. (B) When the red zone reached the colony of *Streptomyces griseus* its growth was promoted at 10 dpi. (C) Growth of *S. griseus* at 13 dpi. (D) No change in the pH of the medium in a single culture of *S. griseus* at 13 dpi. The black triangle indicates the point of inoculation of *S. griseus* .

To verify whether *P. oryzae* can change the extracellular pH, the PDA medium was supplemented with phenol red. When the pH of the medium increased, the colour of the medium changed from yellow to red (Figure 3A). pH around the *P. oryzae* colony increased over time (Figure 3B,C), whereas, in a single culture of *S. griseus*, *the* pH of the medium did not change (Figure 3D). In addition, when the red area reached the *S. griseus* colony, the growth of *S. griseus* was promoted (Figure 3B,C).

### No growth induction with medium removal between P. oryzae and S. griseus

**FIGURE 4:**
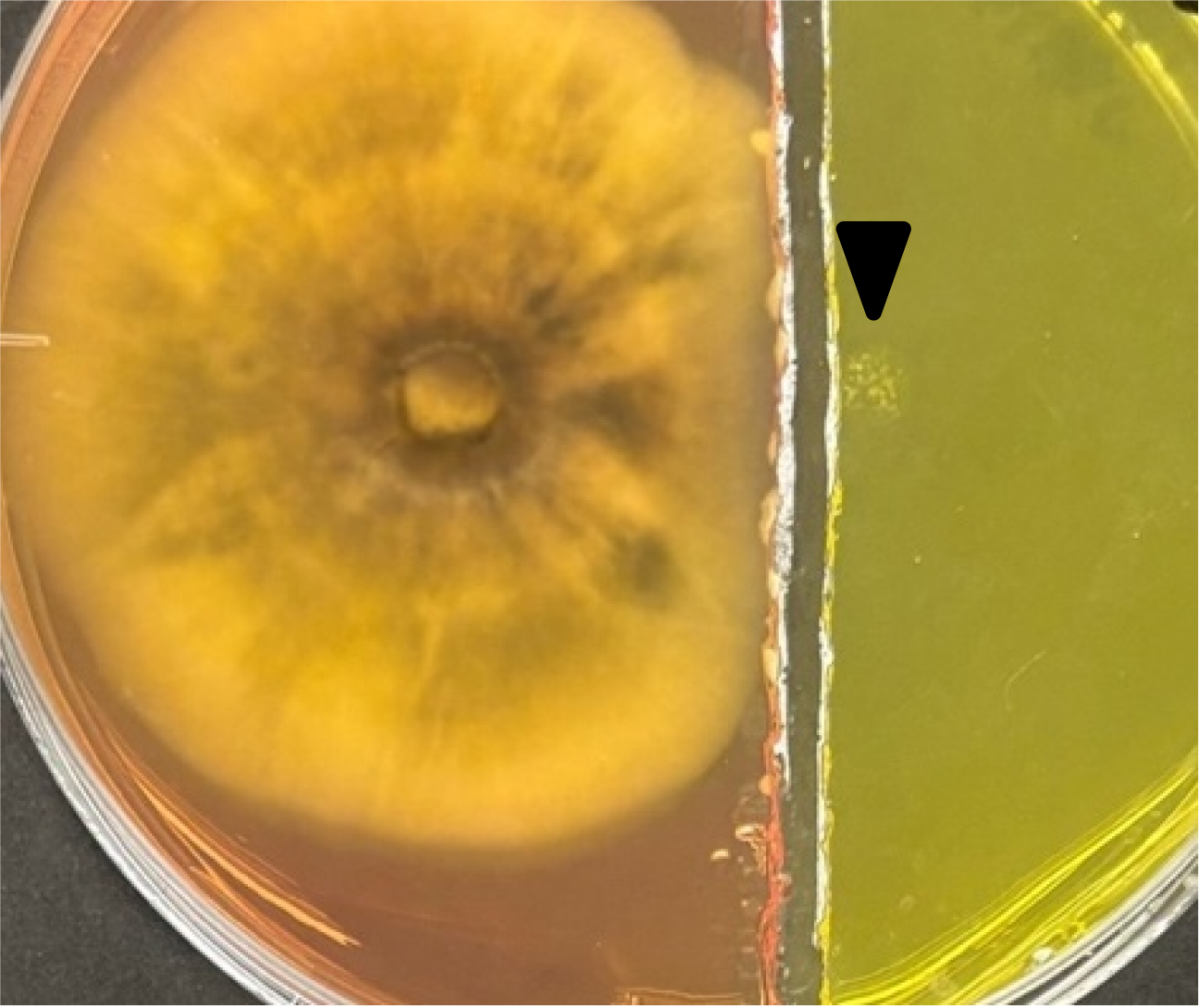
Photograph showing the growth of *Pyricularia oryzae* and *Streptomyces griseus* on separated PDA. No growth of *S. griseus* and pH increased at 13 dpi when growth media were separated. The black triangle indicates the point of inoculation of *S. griseus* .

*S. griseus* growth was not induced when the medium between the growth zone of these two microorganisms was removed, even at 10 dpi (Figure 4). *P. oryzae* increased the pH of the medium regardless of the co‐existence of *S. griseus*. However, the pH of the separated area did not increase (Figure 4). These results suggest that *P. oryzae* produced non‐volatile alkaline compounds, and its production was independent of induction by *S. griseus*.

### Other isolates of P. oryzae can increase pH, enhancing the growth of S. griseus

**FIGURE 5:**
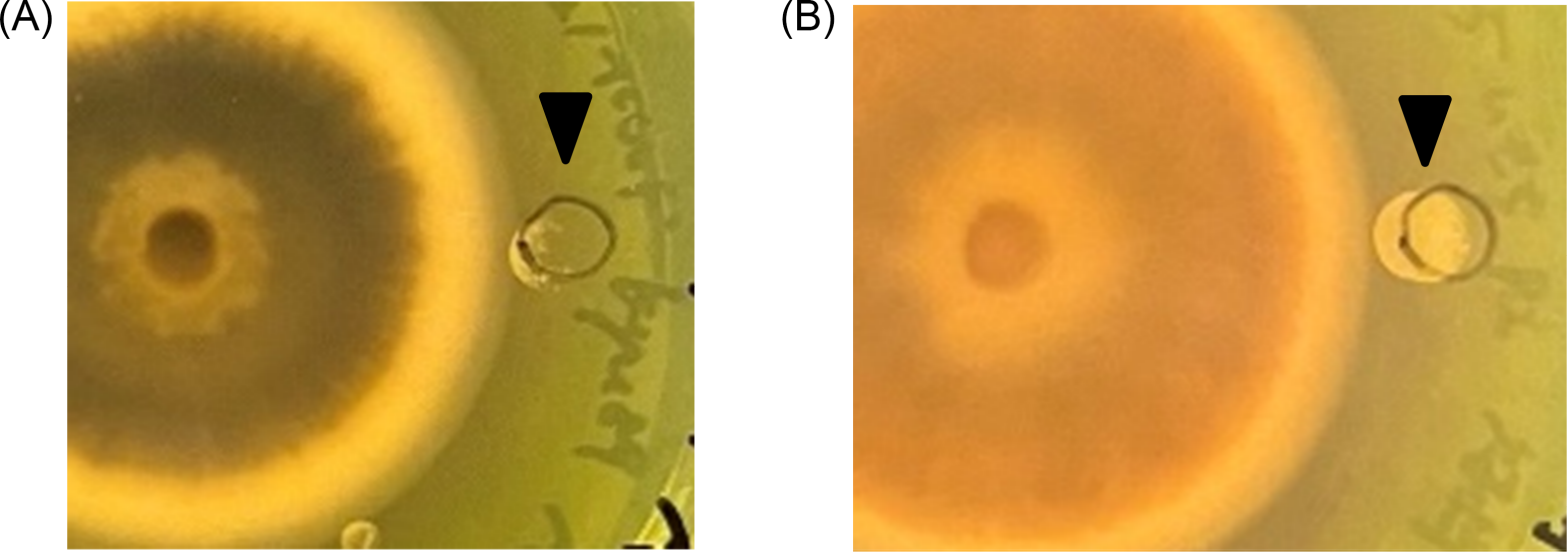
Photographs showing the confrontation culture using other isolates of *Pyricularia oryzae*. Mycelial pictures of the (A) Kyu89‐246 isolate of *P. oryzae* at 7 dpi; (B) P2 isolate of *P. oryzae* at 8 dpi. The black triangle indicates the point of inoculation of *Streptomyces griseus* .

Confrontation cultures were grown using different isolates of *P. oryzae* (Kyu89‐246 and P2). These isolates also changed the pH of the medium and showed a growth‐inducing effect (Figure 5).

### F. oxysporum *and C. tenuipes do not promote the growth of S. griseus nor increase the pH of the medium*

**FIGURE 6:**
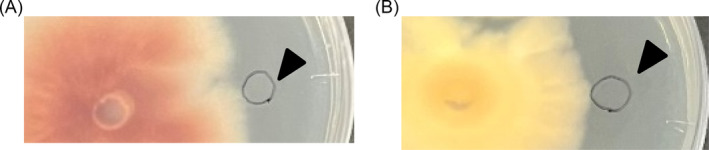
Photographs showing the confrontation culture using (A) *Fusarium oxysporum* and (B) *Cordyceps tenuipes*. Images were captured at 7 and 29 dpi for *F. oxysporum* and *C. tenuipes* , respectively. The black triangle indicates the point of inoculation of *Streptomyces griseus* .

To test whether other pathogenic fungi show similar growth‐promoting potential, the soilborne plant pathogenic fungus, *F. oxysporum*, and an insect pathogenic fungus, *C. tenuipes*, were cocultured with *S. griseus*. These fungi did not promote *S. griseus* growth (Figure 6). This result indicates that growth‐promoting potential is not widely observed in all pathogenic fungi, but is specific to *P. oryzae*.

**FIGURE 7:**
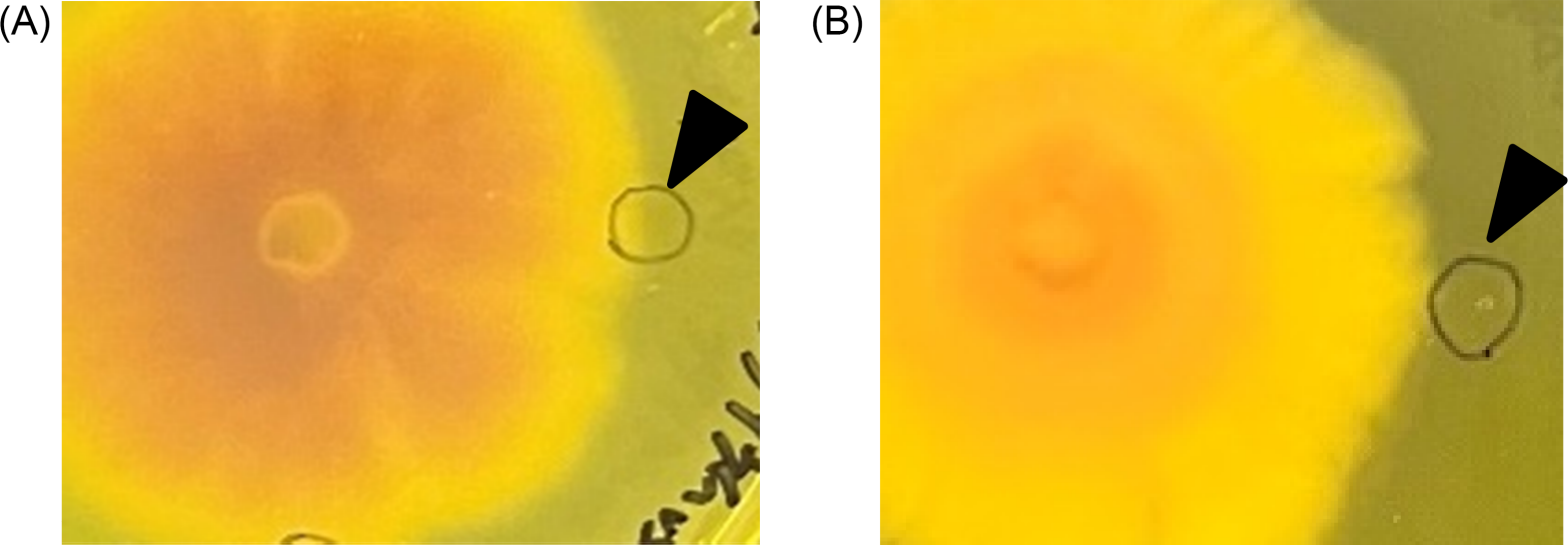
Photographs showing the confrontation culture using *Fusarium oxysporum* and *Cordyceps tenuipes* using phenol red in the medium. (A) *F. oxysporum* at 7 dpi and (B) *C. tenuipes* at 29 dpi do not change the pH of the medium. The black triangle indicates the point of inoculation of *Streptomyces griseus* .

Confrontation culture using phenol red in the medium showed no increase in pH of the medium by *F. oxysporum* and *C. tenuipes* (Figure 7). These results strongly support the fact that the metabolites produced by *P. oryzae* increase the pH of the medium and induce the growth of *S. griseus*.

### Ammonia is not the target compound

**FIGURE 8:**
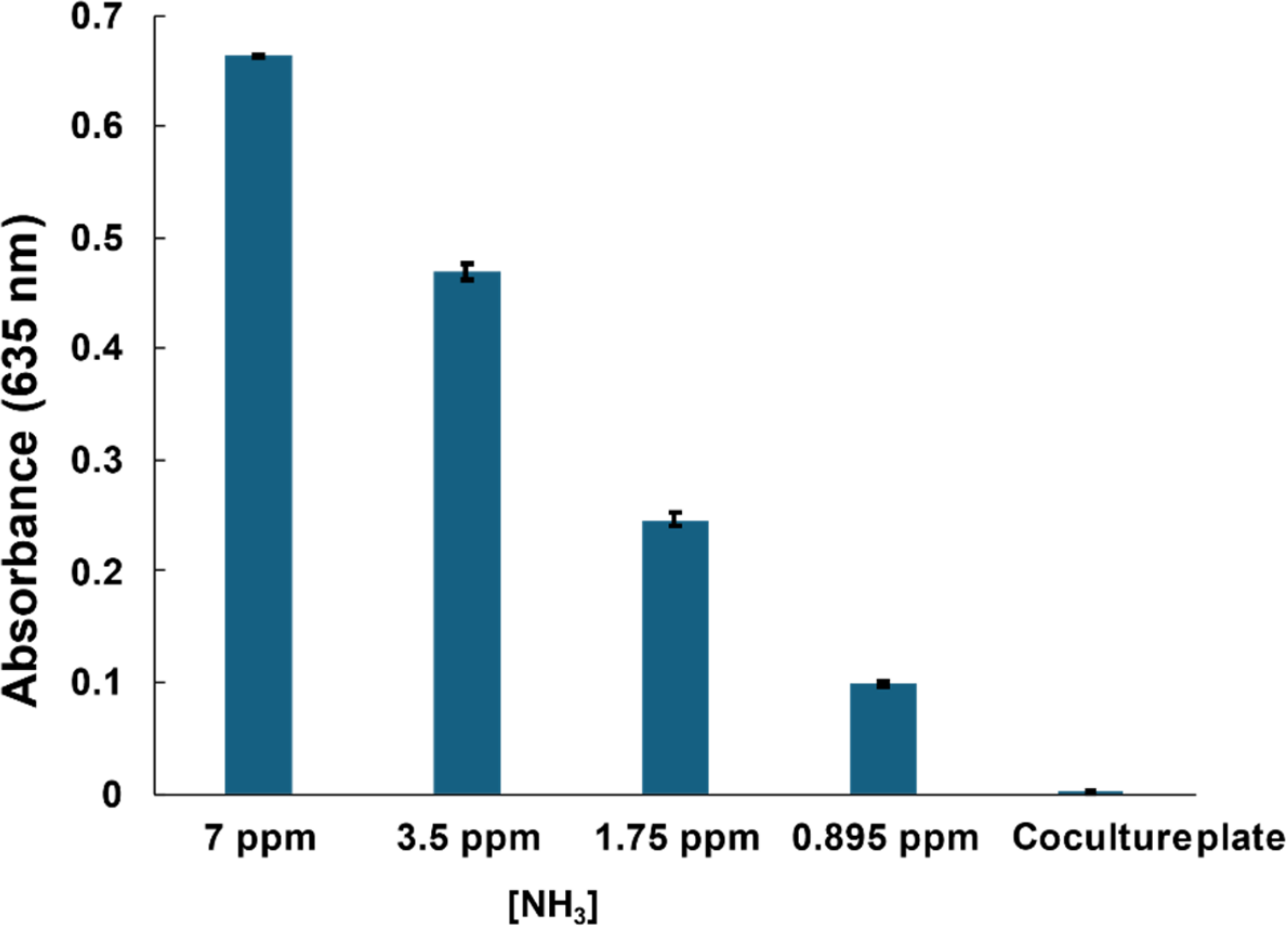
Ammonia detection using the indophenol blue method. The absorption at 635 nm using a UV–vis spectrometer for indophenol blue was measured. The absorbance of ammonia standard solutions (7, 3.5, 1.75, and 0.895 ppm) and the samples extracted from coculture plates (Coculture plate) are indicated. Ammonia was not detected from coculture plates; bars: Standard deviation.

Ammonia was considered a potential compound responsible for increasing the pH of the medium (Fernandes et al., 2017; Landraud et al., 2013; Vylkova, 2017). However, ammonia was not detected in the samples extracted from the plate in which the growth of *S. griseus* was promoted by *P. oryzae* (Figure 8). This indicated that the active compound produced by *P. oryzae* was not ammonia.

### DMFO limited the pH increase of the medium and growth promotion of S. griseus

**FIGURE 9:**
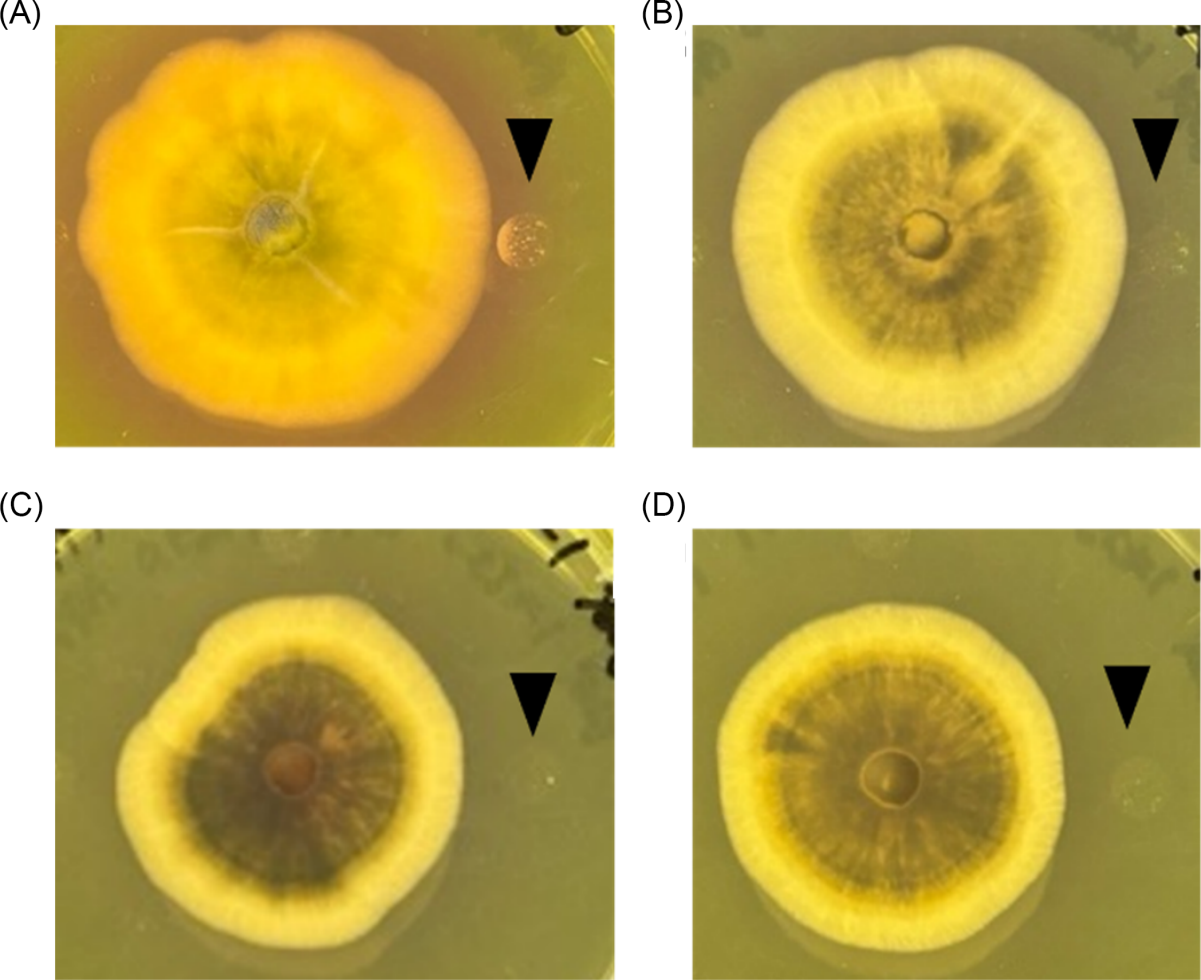
DMFO inhibited the increase in pH and *Streptomyces griseus* growth promotion. DMFO was treated to co‐culture plate of *Pyricularia oryzae* Hoku‐1 and *S. griseus* with (A) 0 mM (non‐treated); (B) 0.1 mM; (C) 0.5 mM; (D) 1 mM. Images were captured at 10 dpi. The black triangle indicates the point of inoculation of *S. griseus* .

DL‐α‐difluoromethylornithine (DMFO), an irreversible inhibitor of ornithine decarboxylase, which is a key enzyme of polyamine biosynthesis, was added to the coculture plate. DMFO inhibited both the rise in pH and growth promotion of *S. griseus* (Figure 9). This suggested that active compound(s) are polyamine(s).

## DISCUSSION

*P. oryzae* causes extensive damage to rice plants (Fernandes et al., 2017). Owing to issues, such as the emergence of drug‐resistant strains, developing new controlling strategies that do not rely on conventional antimicrobial agents is desirable (Kunova et al., 2021). In recent years, new methods of microbial control based on microbial interactions have attracted considerable interest (Pierce et al., 2021). This study may be useful for developing new methods for controlling rice blast with minimal environmental impacts. Furthermore, reports on the ability of competitive microorganisms to promote their growth are very less. To the best of our knowledge, this is the first report that *P. oryzae* promote the growth of actinomycetes in a non‐contact manner. Therefore, this study is unique in terms of microbial ecology.

The results of this study suggested that the growth of *S. griseus* was promoted by an increase in the pH of the medium caused by the compounds produced by *P. oryzae*. Phytopathogenic fungi produce ammonia during growth, and the pH of the environment increases (Fernandes et al., 2017; Landraud et al., 2013; Vylkova, 2017). Therefore, ammonia was considered to be a growth‐inducing factor in the present study. However, ammonia was not detected in the medium in which actinomycete growth was induced by confrontation culture. Therefore, ammonia was not involved in this phenomenon of growth induction. Trimethylamine, a volatile polyamine, produced by *Streptomyces venezuelae* promotes its own growth by increasing the pH of the medium (Jones et al., 2017; Shepherdson et al., 2023). When cocultured with *Saccharomyces cerevisiae*, the growth of *S. venezuelae* is stimulated, and trimethylamine is produced. Therefore, the substance(s) responsible for the growth induction of *S. griseus* in the present study may be polyamine(s). Trimethylamine has been reported to affect the growth of *S. venezuelae* in distant locations since it is a volatile compound (Jones et al., 2017). However, in the present study, growth induction was not observed when the medium between *P. oryzae* and *S. griseus* was removed, suggesting that the active compounds diffused through the medium without volatilization. Furthermore, it is also reported that the growth of *S. griseus* is not affected by *S. cerevisiae* (Jones et al., 2017). These results indicated that the mechanism of growth induction of *S. griseus* by *P. oryzae* was different from those in previous reports. Other polyamines, such as spermidine, spermine and putrescine, are produced by *P. oryzae* (Subba et al., 2022). These polyamines may be responsible for the growth induction of *S. griseus*.

It was reported that a rise in pH during *P. oryzae* infection along with ammonia production which correlated with the rise in pH of the liquid medium during growth of this fungus (Landraud et al., 2013). However, to successfully alkalinize and kill plant tissue by ammonia production, fungi must first grow enough hyphal biomass in mildly acidic plant tissue (Fernandes et al., 2017). In addition, in the early stage of infection, *P. oryzae* grows with biotrophic behaviour (Landraud et al., 2013). *P. oryzae* may adapt to the ambient environment in plant tissue through neutralization without killing the host plant, as reported in this study.

Both *F. oxysporum* and *C. tenuipes* inhabit soil (Sung et al., 2007; Yan et al., 2023). *S. griseus* is also found primarily in soil (Fischer et al., 2018); therefore, *F. oxysporum* and *C. tenuipes* may encounter *S. griseus*. However, these fungi did not promote the growth of *S. griseus*. This suggests that the phenomenon is specific to *P. oryzae*. Notably, *P. oryzae* is less abundant in soil (Marcel et al., 2010). This led us to propose two possible interaction patterns; either *P. oryzae* is present in soil or it interacts with *S. griseus* in rice plants. Because the same phenomenon was observed with *P. grisea*, *S. griseus* may be endophytic in Poaceae plants and interact with *P. oryzae* in rice. Other endophytic *Streptomyces* spp. in rice plants are useful in controlling blast fungi (Law et al., 2017).

We have previously reported a compound‐mediated competitive relationship between *S. griseus* and *P. oryzae* (Furuyama et al., 2021). At first glance, the results of the present study appear to contradict the previous report. This phenomenon may be a competitive response of *S. griseus*, rather than an active growth induction by *P. oryzae*. Namely, *S. griseus* can detect the presence of *P. oryzae* before contact with *P. oryzae* and *S. griseus* grows sufficiently to produce antibiotics. Antibiotics from *S. griseus* induce the production of secondary metabolites in *P. oryzae* (Furuyama et al., 2021). *S. griseus* first produces an antibiotic, and *P. oryzae* senses it and produces secondary metabolites to counteract *S. griseus*. Therefore, *P. oryzae* and *S. griseus* may share a common niche, possibly in rice plants, and maintain a competitive relationship. *S. griseus* have potential as a new biocontrol agent for *P. oryzae* with minimal environmental impacts.

## CONCLUSION

*S. griseus* growth was promoted by *P. oryzae* owing to the increase in pH of the medium caused by non‐volatile substances produced by *P. oryzae*. Furthermore, this is the first report of *Pyricularia oryzae* neutralizing the environment by producing a non‐volatile compound. These results shed light on partial ecology to pH modulation of *P. oryzae*, which is observed in the biotrophic infection stage, in a laboratory setting.

## AUTHOR CONTRIBUTIONS

**Risa Sugiura:** Writing – original draft; methodology. **Takayuki Arazoe:** Writing – review and editing. **Takayuki Motoyama:** Writing – review and editing. **Hiroyuki Osada:** Writing – review and editing. **Takashi Kamakura:** Writing – review and editing. **Kouji Kuramochi:** Writing – review and editing; supervision. **Yuuki Furuyama:** Writing – review and editing; conceptualization; project administration.

## CONFLICT OF INTEREST STATEMENT

The authors declare no conflicts of interest.

## Data Availability

The data that support the findings of this study are available from the corresponding author upon reasonable request.
